# Gender Equality and the COVID-19 Pandemic: Labour Market, Family Relationships and Public Policy

**DOI:** 10.1007/s10272-021-0997-2

**Published:** 2021-10-05

**Authors:** Paola Profeta

**Affiliations:** Department of Social and Political Sciences, Bocconi University, Via Roentgen, 1 (3rdfloor), 20136 Milano, Italy

## Abstract

During the recovery, investing in gender equality is essential: it will lead directly to higher GDP and indirectly to increase human capital and promote a sustainable society.

Or Women as economic agents may themselves have an impact on policies: the changing role of women in families and societies and their greater representation in decision making positions contribute to focusing and redirecting the policy agenda toward items that ultimately reduce gender gaps.

The COVID-19 pandemic has affected the lives of women and men around the world. The coronavirus crisis is not only a health crisis, but also an economic one, with severe consequences for the labour market. The pandemic has deepened pre-existing inequalities, particularly gender inequality. Even if regional variations exist, evidence suggests that women have generally been more vulnerable to social and economic risks and harder hit by the consequences of the pandemic (see for instance, Profeta, 2020a; Profeta, 2020b; Alon et al., 2020).

The pandemic has exacerbated pre-existing gender gaps, resulting in the emergence of the "she-cession". In contrast to previous economic recessions, such as the 2008 financial crisis, the service sector – where women are disproportionately employed – has been hardest hit by lockdown and social distancing measures. Moreover, living conditions and work-life balance have been seriously affected, since housework and childcare have increased during the pandemic. Women have taken on a disproportionate amount of uncompensated childcare work, even if enforced lockdowns have meant that men have increased their household participation in comparison to the years prior to the pandemic. This re-arrangement of family relations represents an opportunity for change in the future in which household and childcare tasks could become more equally divided and thus permit women to increase their participation in the labour market.

However, the increased housework and childcare burden has so far fallen mainly on women, thus posing an additional challenge to gender inequalities. This is particularly strong in countries where conservative gender roles in the family are well established and the cultural context does not promote gender equality due to persistent stereotypes and biases. Overall, in the absence of targeted policies to promote gender equality, the progress achieved to date is at risk of being rolled back.

This paper examines the main dimensions of gender inequalities before the pandemic and their evolution during the COVID-19 crisis. It focuses on Europe and identifies three different main interrelated dimensions: labour market, family relationships – namely, how COVID-19 affected the unpaid care burden and living conditions of working parents – and public policies that could help achieve a more gender-equal recovery.

## The labour market

Labour market data show persisting gender inequalities around the world. In Europe, women are more educated today than men – the share of female graduates is higher in almost all European countries. Although differences persist in the fields of study, with women less present in STEM (science, technology, engineering, mathematics) disciplines, closing the gender gap in education is recognised as a first step towards gender equality in the labour market (see Profeta, 2020a). Yet in all countries, the female employment rate is lower than that of males.

Figure 1 reports male and female employment rates in European countries before the pandemic for people aged 20-64 years. The female employment rate differs across countries: While the average female employment rate in Europe in 2019 was 68.2% (the corresponding value for males was 80%), countries such as Italy and Greece lag behind with only slightly above 50%, while Sweden was closer to 80%. Entering the pandemic with a different initial level has contributed to different impacts of the crisis.

There are other indicators that also play a role: The gender pay gap is substantial (according to Eurostat, 2021), it amounts on average to 16% in the EU), especially in countries where the female employment rate is higher; the motherhood or child penalty is sizeable (Kleven et al., 2019); the obstacles for establishing a career – also called the glassceiling – are a real phenomenon, resulting in notably fewer women in decision-making positions.

Female employees have been disproportionately and negatively affected by the COVID-19 crisis vis-à-vis their male counterparts. Men tend to be employed in highly cyclical sectors (e.g. manufacturing, construction) which are increasingly impacted in regular economic recessions, leading to a greater likelihood of unemployment in an economic crisis for men. During the coronavirus crisis,

Figure 1

**Male and female employment rate across European countries before the COVID-19 pandemic**

percentage of the population aged 20-64, 2019

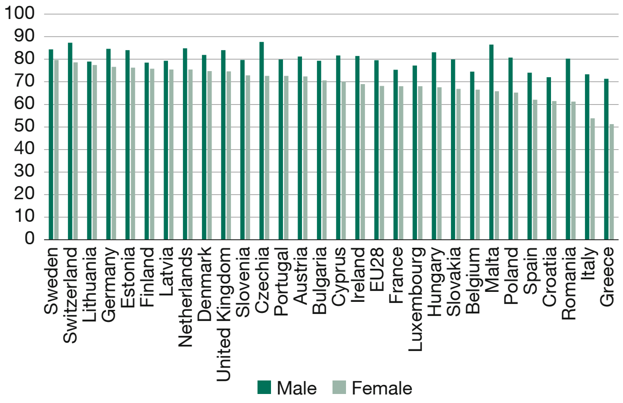

Source: Author's elaboration of Eurostat data.

however, the service sector has been the most affected due to the consequent lockdown and social distancing measures. As women are disproportionally employed in the service sector, a "she-cession" has emerged. Differently from the previous economic recessions, such as the 2008 financial crisis, the governmental measures to halt the pandemic have had the most indirect impact on the economic sectors in which women tend to be overrepresented, including hospitality, gastronomy, retail, care and domestic work (Alon et al., 2020).

Women have disproportionately lost their jobs at the onset of the pandemic and they have also encountered greater obstacles to re-enter the labour force in the period between the first two waves of COVID-19 cases in the summer 2020, when Europe experienced a partial economic recovery. That is, while employment prospects rose by 1.4% for men, they increased by a mere 0.8% for women between the second and third quarter of 2020 Eurostat (2020).

The risk of poverty has also increased. Not all women have been equally affected. Apart from cross-country differences, women are also intersectionally divided according to other demographic pillars. Lower-income and lower-skilled women tend to encounter themselves in a vicious cycle of systematic poverty. Older women and single women now also face a stronger likelihood of poverty (Calò et al., 2021).

## Family relationships

The COVID-19 pandemic and the consequent lockdown and closure of schools has increased the amount of work for families and reduced the childcare support from grandparents. Several studies (see Del Boca et al., 2020) agree that this additional burden has not been equally shared between men and women within the family but rather has fallen mainly on women, exacerbating pre-existing differences; even before the pandemic, OECD (2021) estimates that on average women spent two hours per day more than men on unpaid work at home.

Even for couples who both worked from home, the amount of housework and childcare has not been equally shared. That was not an obvious result *ex ante*, since working from home may induce more involvement of men in family chores. Yet, pre-existing gaps have exacerbated the ex-post asymmetric outcome. In Italy, one of the countries more seriously hit by the pandemic, Del Boca et al. (2021) have analysed the division of housework and childcare in a sample of 800 representative Italian working women and their partners during the first and the second waves of the COVID-19 pandemic (April 2020 and November 2020). The authors first show that the distribution of the extra family work between women and their partners depends on the work arrangements of each partner during the lockdown (working from home, working outside the home, not working). During the first wave of the pandemic, 44% of women and 30% of their partners worked from home. The gap remained during the second wave of the pandemic (24% versus 15%). However, even within the couples where both partners worked from home, women increased their daily hours of housework from 2.52 hours before the pandemic to three hours during the first wave on average, while men increased their daily hours of housework from 1.26 to 1.57. The gender difference remained significant, though smaller, even during the second wave of the pandemic (2.82 hours for women versus 1.43 for men). Childcare is more equally shared between parents, though the difference between men and women remains significant. Similar results are obtained, among others, by Biroli et al. (2020) for Italy, UK and US; Farré et al. (2020) for Spain; Sevilla and Smith (2020) for the UK.

The consequences of the asymmetric division of housework and childcare within couples may be enormous, not only on the labour market but also on fertility rates: Mothers are more likely to be employed full-time and to have an additional child when fathers are more involved in the family (Fanelli and Profeta, 2021).

## Public policies

Policies play an important role in the promotion of gender equality, including policies to support women's entrance into the labour market, to remain in the labour market after childbirth and to develop their careers equally to men. Public policies include childcare as well as paternity leave, which supports a more equal balance of housework and childcare between men and women. More balanced gender roles starting with the family will be reflected in the labour market and firms' views. These policies, which are publicly based but also privately provided, are shown to support not only female employment but also fertility rates, which are declining in OECD countries, thus creating a positive virtual equilibrium for growth.

During the pandemic of COVID-19, several countries have invested in policies directed at families. These measures are meant to support family needs and their work-life balance. They provide additional time for parental leave or in-kind benefits (baby-sitter vouchers, allowances) in particular during the lockdown and upon closure of schools and childcare providers. These measures are also meant to support working women (see Profeta, 2020c for a map of family policies implemented as a response to COVID-19). Indirect effects on gender equality are also expected from the diffusion of flexible work arrangements, which have accelerated during the pandemic. In fact, working flexibly in time and space may have the effect of increasing the involvement of men in housework and childcare (Angelici and Profeta, 2020), thus helping to rebalance the asymmetric allocation of family chores within the couple.

Investing in policies that support gender equality goes beyond ensuring equal opportunities between men and women. It is a valuable investment with multiple returns for the economy. In fact, gender equality and women's empowerment are associated with economic growth and development, sustainable growth and a better use of talents. During the recovery, investing in gender equality is essential: It will lead directly to higher GDP and indirectly to increasing human capital and promoting a sustainable society (Profeta, 2020a). This strategy is in line with the UN development goals, namely goal number five, which aspires "to achieve gender equality and empower all women and girls" as a necessary foundation for a peaceful, prosperous and sustainable world.

A major concern for working women during the pandemic has been the closure of schools and childcare facilities. When schools and nurseries close, the amount of childcare correspondingly increases. In households with non-working mothers, they may be expected to bear most of the additional childcare responsibilities. Maternal employment instead is seriously affected. Interestingly, there is a strong negative correlation between the maternal employment rate (before the pandemic) and the share of school closing days during the pandemic (Calò et. al, 2021). In countries with lower maternal employment, policymakers tend to be keener on closing schools, as they may perceive lower organisational costs to the families. However, this may also reinforce the reduction of maternal employment, with negative long-run effects on gender equality.

Not only do public policies drive gender equality, but gender equality can also reinforce public policies. Another important lesson from research about the beneficial effects of gender equality is that gender-balanced leadership (in business and politics) can be beneficial (Profeta, 2020a). Women as economic agents may themselves have an impact on policies: The changing role of women in families and societies and their greater representation in decision-making positions contribute to focusing and redirecting the policy agenda towards items that ultimately reduce gender gaps. In fact, women change the agenda by giving more weight to expenditures in family and early childcare, education and social issues, which in turn increase gender equality. Moreover, women leaders may act as role models for cultural change, which also boosts gender equality. Women leaders contribute to a better selection of decision-makers and to overall performance – we know this from research on gender quotas for company boards: Gender-balanced leadership increases the average quality of the leaders (Ferraro et al., 2021) with positive effects on performance. Female leaders also contribute to an inclusive style of leadership and a more inclusive agenda, where sustainability and diversity are a key goal.

The pandemic has provided evidence about the value of female leadership. The evidence is still anecdotal, but it shows that countries led by women – such as Denmark, Finland, Germany, Iceland, New Zealand, Norway and Taiwan – have experienced better performance during the pandemic than those led by men. Obviously, a simple correlation does not imply causality. Yet some traits of the female leadership have emerged during the pandemic: honesty, determination, inclusion, innovation in the use of technology and communication and information.

Interestingly, in all countries women have been more respective of rules than men (Galasso et al., 2020). They have been more concerned by the effects of the pandemic and ready to follow the rules, such as wearing masks, reducing mobility, closing activities, quarantine and lockdown.

## Conclusions

In spite of the constant and continuous progress towards gender equality, no country in the world has achieved it. Economic opportunities and political empowerment are the more critical dimensions (World Economic Forum, 2021). The pandemic poses the serious threat of exacerbating existing gender gaps and enlarging inequalities, especially in countries where these were highly pronounced before the COVID-19 outbreak. We have already seen data of declining female employment during 2020. The asymmetric burden of increased housework and childcare has added a further push to the “she-cession”. The increased incidence of violence, particularly intimate-partner violence, denominated the “shadow pandemic”, also requires specific policy interventions.

Public policies may play an important role in supporting a “she-covery”, based on the emergence of a virtual process: investing in policies to promote female employment and supporting gender-balanced leadership in policymaking in order to design successful strategies and reduce inequalities.
